# Study on Tensile Properties of Fly Ash, Sugarcane Fiber and Carbon Nanotube-Reinforced Polymer Matrix Composite Using Objective Evolutionary Algorithm

**DOI:** 10.3390/nano12234112

**Published:** 2022-11-22

**Authors:** Gopalan Venkatachalam, Arunkumar Gopu, Pitchumani Shenbaga Velu, Neelanarayanan Venkataraman, Dinesh Ramesh Salunke, Raghava Rao Mukkamala

**Affiliations:** 1 Centre for Innovation and Product Development, Vellore Institute of Technology, Chennai 600127, India; 2 School of Computer Science and Engineering, VIT-AP University, Amaravati 522237, India; 3 School of Mechanical Engineering, Vellore Institute of technology, Chennai 600127, India; 4 School of Computer Science and Engineering, Vellore Institute of Technology, Chennai 600127, India; 5 Department of Digitalization, Copenhagen Business School, 2000 Frederiksberg, Denmark

**Keywords:** natural fibers, carbon nanotubes, ANOVA, mechanical properties, MOEA/D algorithm

## Abstract

Composite materials have a wide range of applications in emerging eco-friendly environments. Composites that created from naturally available materials are easily decomposed over time and very cost-effective. Fly ash and sugarcane fiber are widely available waste materials produced on a massive scale. This research was aimed to find an optimal mixture of reinforced composites (fly ash, sugarcane fiber and CNTs) in order to maximize yield strength, ultimate tensile strength and Young’s modulus using a Multi-Objective Evolutionary Algorithm with Decomposition (MOEA/D). Optimizing one objective may have a negative impact on another objective, so the authors used the sophisticated MOEA/D algorithm to simultaneously find optimal values on all three objectives. The Design of Experiments (DOE) method was performed using ANOVA, and then regression equations were generated. The regression equations were optimized using the MOEA/D algorithm to obtain optimal values. Using the optimal compositional values produced by the algorithm, materials were fabricated. The fabricated materials were tested using a Shimadzu UTM machine to cross-validate the findings. A combination of 0.2 wt.% of fly ash, 2 wt.% of SCF, and 0.39 wt.% of CNTs showed a maximum yield strength of 7.52 MPa and Young’s modulus of 1281.18 MPa, with a quite considerable ultimate tensile strength of 10.54 MPa compared with the optimized results obtained through the response surface methodology.

## 1. Introduction

In the last two decades, lightweight materials have played a vital role in improving mechanical strength. Composites are substances that have been mechanically or metallurgically bound together to combine the advantageous features of many materials. Although each component’s structure formation and characteristics are preserved, a composite often has superior qualities. Due to their high stiffness, strength and resistance to wear, composite materials outperform traditional alloys in a variety of applications. They are generally applied in various manufacturing components, such as buyer products, electrical rods, and shipping accessories [1,2,3,4]. In recent years, much research has been carried out to enhance the mechanical properties of composites. For single- and multi-response process optimization, researchers have used a variety of methodologies, including response surface methodology, neural networks, the Gray algorithm, the genetic algorithm, and particle swarm optimization. Process performance is influenced by the optimal setting of the process parameters. It is required to evaluate the impact of each process parameter on each response parameter for multi-response optimization [5].

Optimization has left its footprint in various engineering domains. Optimization is the rigorous search of a solution space to attain a better solution. Brute force is a technique that searches every possibility in a search space, whereas a heuristics-based algorithm uses a guided random approach to effectively search for a solution in less time [6]. Bio-inspired optimization algorithms have been applied to many leading industrial problems in the continuous and discrete domains. Optimization problems are generally classified into two types, i.e., single-objective optimization and multi-objective optimization. In single-objective optimization, the algorithm only considers one objective function and the output of the algorithm is a scalar quantity. In a multi-objective optimization problem, more than one objective function is mapped in the algorithm [7]. The output of a multi-objective optimization problem is usually presented in vectors. Each value in the output vector corresponds to an objective function. The objective function or fitness function is the mathematical model constructed specifically for the problem instance that is to be maximized or minimized with the aid of optimization algorithms. The search space comprises all the possible inputs given to an algorithm to produce an output in the solution space. The search space can be minimized using constraints. Every optimization algorithm has three working phases, namely: initialization, iteration, and the identification of the global best solution [8]. In the initialization phase, the parameters required for the algorithm, the objective function, and the exploration and exploitation weight vectors are initialized. The initial problem-specific population is generated at random and evaluated using the objective function. Exploration is the process of searching the entire search space, whereas exploitation is a way to improve the existing solution. Every bio-inspired algorithm is designed to run for a number of iterations. The weight vectors are coefficients that increase or decrease the exploration and exploitation as the iterations proceed. During the initial iteration, exploration is given more weightage, and as the iteration increases, the exploration coefficient becomes 0 or minimal and exploitation is given more weightage. If the exploration coefficient is initialized to a constant, then the algorithm works as a random walk. The initial population is improved in the iteration phase. The initialization phase remains the same for most bio-inspired algorithms [9]. The iteration phase is the one that differentiates a variety of bio-inspired algorithms. For example, the ant colony algorithm uses a different iteration phase compared with the genetic algorithm. In this phase, unique methodologies are used to improve existing solutions. During each iteration, the best of all solutions is stored in an elite population, and it is replaced if any better solutions are found [10]. 

For a single-objective optimization problem, the identification of a global solution is conducted by simply comparing the objective values of a resultant population with the global best values. This phase varies in the case of multi-objective optimization problems [11]. Naik et al. [12] used the genetic algorithm (GA) to achieve global optima to minimize the weight of a carbon epoxy composite laminate rather than using gradient descendant-based techniques. The native reproduction, crossover and mutation operators were used. It was observed that the gradient-based methods could not be used to find global minima when a local minimum was found. Gillet et al. [13] implemented a multi-objective version of the GA to optimize two objective functions to study the influence of design variables in various standard optimization problems. The adoption of strength a Pareto evolutionary algorithm was implemented by Zitzler and Thiele [14] to isolate non-dominated solutions in an entire population. Almeida and Awruch used a modified version of a reproduction and crossover operator to optimize composite laminated structures. Minimizations of the weight and cost of materials were simultaneously addressed through multi-objective optimization. Single-point crossover was used as the reproduction operator. The random addition or subtraction of values under limits and gene swapping were used as mutation operators to ensure diversity among the population [15]. 

Irisarri et al. [16] used GA-based multi-objective optimization for the buckling of laminated plates. The initial population was initialized with the values obtained from the maximin design of experiments method to speed up the convergence rather than using a randomized initial population. Spears and Anand [17] used two-point crossover as a reproduction operator. Sheyka et al. [18] used a multi-objective genetic algorithm (MOGA) to design a blast-resistant composite. Murata et al. [19,20] adapted the GA to address multiple objectives using weight vectors and summated the objective score to a single value (weighted sum approach) Alvarez et al. [21] used a MOGA in which the optimization was constructed without the need of merging the solution by using the concept of domination. A MOGA extensively searches a solution front to produce multiple Pareto solutions, whereas the weighted sum approach can only be used identify a solution over the imposition of weight vectors. Lee et al. [22] presented a work that aimed to minimize the weight of multilayered composite plates and minimize their maximum displacement. The design variables included the type, thickness and orientation of the fibers of each layer. Duk et al. [23] evaluated objective and constraint functions instead of using time-consuming finite element analysis methods (FEAMs) during the optimization process, while the NSGAII was employed to find a set of Pareto-optimal solutions of MOO problems. Additionally, the effects of various boundary conditions and carbon nanotube (CNT) distributions on the Pareto-optimal solutions of MOO problems were discussed. Toupe et al. [24] investigated the effect of two different optimization paths on the microstructure and mechanical properties of flax fiber/postconsumer recycled plastic composites.

Badallo et al. [25] compared the performance of the archive-based micro genetic algorithm (ABGM), neighborhood cultivation genetic algorithm (NCGA) and non-dominate sorting genetic algorithm II (NSGA-II) to maximize the critical buckling load and minimize the mass. Tiwari et al. stated that the ABGM is an improved version of the GA, as the history of an entire population can be stored in an external archive. Once executed, the best-performing individuals form the Pareto front. Deb et al. [26,27] confirmed that the NCGA follows the same flow as the GA, except that crossover is performed between individuals of closest objective values. Diversity was achieved using extensive mutation operators. Munck et al. [28] stated that in the NSGA II, the concept of domination is applied to parent and children populations, and then multiple Pareto fronts are identified to perform crowding distance calculations. An individual lesser crowding distance denotes the extensive availability of solutions. The NSGA II only maintains an external archive with Pareto solutions. The external archive is replaced during every iteration with a better performing solution. Hwang et al. [29] used a roulette wheel method to select an individual to be included in a mating pool in which the best-performing individual has a higher probability of being including in the mating pool than the worst-performing individual. In this article, the authors used a layer-wise optimization algorithm (ILOA) to design viscoelastic composite structures.

Gopalan et al. [30] studied the impact of natural fiber content on ultimate tensile strength. It was observed that fly ash did not affect UTS, but the addition of carbon nanotubes (CNTs) enhanced other mechanical properties. Gopalan et al. [31] studied the tensile properties of natural fiber/carbon nanotubes reinforced with EC. Samples were prepared using the Design of Experiments (DOE) approach with the CCD of the RSM, and then the effect of the wt.% of each constituent on tensile properties was analyzed with the ANOVA model. It was observed that YS and YM mainly depended on the wt.% of sugarcane fiber, whereas fly ash and CNTs contributed to the enhancement of the ultimate tensile strength. The yield strength, ultimate tensile strength, and Young’s modulus of a composite of 0.5 weight percent fly ash, 2 weight percent natural fiber and 0.85 weight percent carbon nanotubes were determined to be 5.53 MPa, 19.62 MPa, and 914.96 MPa, respectively. Sim et al. [32] evaluated the tensile strength of a fly ash/epoxy-reinforced polymer matrix composite for different vol.% of fly ash. It was observed that the tensile strength increased with increases in the content of fly ash to certain limit and then decreased. 

Khondker et al. [33] considered composite specimens produced under various processing parameters. Under the identified suitable processing parameters, jute/PP composite samples exhibited excellent mechanical characteristics. When compared with virgin PP materials, the unidirectional jute/PP composites’ static mechanical characteristics were significantly improved.

The effects of compression process parameters on the tensile properties of composites reinforced with hemp fiber were investigated by Takemura and Minekage [34]. They discovered that compared with polypropylene bulk, hemp fiber-reinforced polypropylene had better tensile properties. In comparison with the resin large bulk specimen, the composite was 2.6 times stronger. The ideal molding temperature and time for hemp fiber-reinforced green composite were found to be under 180 °C and 20 min, respectively. Goleswski et al. [35] analyzed increases in the early strength of concrete with fly ash through the application of a specifically formulated chemical nano-admixture (NA) in the form of seeds of the C-S-H phase. The NA was used to accelerate the strength growth in the concrete. The results of tests indicate the possibility of using NA in a wide range of management areas in sustainable concrete prefabrication. Khan et al. [36] noted that the application of nano-silica in cement-based composites was beneficial when used up to an optimal dosage of 2–3% due to high pozzolanic reactivity and a filler effect, whereas a higher dosage of nano-silica had a detrimental influence due to the increased porosity and microcracking caused by the agglomeration of nano-silica particles. The mechanical strength could be enhanced by 20–25% when NS was incorporated in the optimal amount. The models developed for predicting the strength of nano-silica-modified concrete exhibited good agreement with experimental data according to low error values. Zhang et al. [37] observed that geopolymer composites’ flowability and compressive strength were slightly improved when compared with those without NS. With the increase in the superplasticizer content, the compressive strength of geopolymer composites showed a slightly decreasing trend on the whole.

Aamir et al. [38] examined the drilling process parameters and their optimization techniques, as well as the effects of dust particles on human health during the machining process. They observed that the direction of fibers had an important role in defining the damage tolerances of composites, chip formation, thrust force, and surface roughness. For the optimization of process parameters, a multi-attribute decision-making technique has been used by different researchers; however, the majority have studied the Taguchi method because it is a simple approach that does not require an expert background in statistics to form a set of standard designs.

From the literature, it can be observed that a few studies have reported the effects of various constitutes on the tensile properties of polymer matrix composites but no one has used a multi-objective evolutionary algorithm to find the optimized wt.% for the mentioned composites. Hence, in this research, the authors attempted to find out the optimum wt.% of sugarcane fiber, fly ash and CNTs to achieve maximum tensile properties using a multi-objective evolutionary algorithm technique that was also validated with the experimental and ANOVA results listed in a previous study carried out by authors. In this research work, the authors employed a multi-objective evolutionary algorithm using decomposition to find an optimal value considering the parameters of yield strength, ultimate tensile strength and Young’s modulus. These parameters were programmatically embedded as an objective function in the MOEA/D algorithm. The algorithm produced an output vector with three values corresponding to each objective function. The concept of domination was used to identify the best-performing solution among the generated solutions. The worst-performing solutions were eliminated using the concepts of domination, the remaining solutions were plotted in a three-dimensional space, and a Pareto front was constructed. All the solutions in the Pareto front were non-dominated or best-performing solutions.

## 2. Methodology

**Figure 1:**
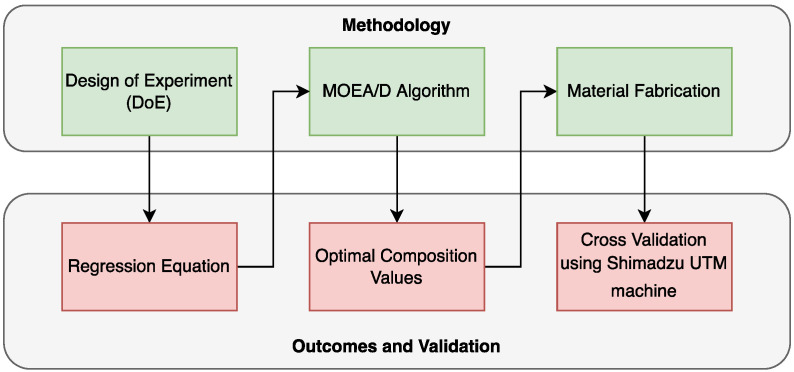
Process flowchart.

A flowchart for the proposed method is shown in Figure 1. In this study, the influences of the wt.% of fly ash, sugarcane fiber and CNTs on tensile behaviors were studied using analysis of variance (ANOVA). The tensile test values were fed to the MINITAB software program to carry out ANOVA. Regression equations, which illustrated the influences of the wt.% of fly ash, sugarcane and CNTs on the tensile behavior of the composite, were obtained. The regression equations were optimized using the MOEA/D algorithm to obtain optimal values. Using the optimal compositional values produced by the algorithm, materials were fabricated. The fabricated materials were tested using a Shimadzu UTM machine to cross-validate the findings. 

### 2.1. Materials and Methods 

The polymer matrix composites were made using epoxy as the matrix. Fly ash was utilized as one of the fillers and reinforcements since it is a plentiful and accessible substance. Fly ash with a grain diameter of 50 μm was purchased to create an epoxy polymer matrix composite. Due to its biodegradability, sugarcane fiber was also used as reinforcement with the matrix. Dried sugarcane was acquired and processed through a pulverizer to create a reinforcement for an EPM composite. The sugarcane fiber was filtered in a sieve with a mesh size of 150 μm after it was ground. As a result, the grain diameter of the sugarcane fiber used in the epoxy polymer matrix composite was 150 μm. A CNT is a substance that considerably raises the malleable property of an EPM composite when used as a filler or reinforcement. MWCNTs purchased from Sisco Research Laboratories Pvt. Ltd., (Chennai, India) were used to add another filler or reinforcement to the epoxy polymer matrix. The purchased CNTs had the following specifications: MWCNT type 3, outer diameter of 10–20 nm, and span of 10–30 μm.

For the preparation of samples, the Design of Experiments (DOE) concept was used to keep track of the variations of all involved factors. Table 1 shows the parameter levels for the wt.% of the fly ash, sugarcane fiber, and CNTs obtained using the RSM model CCD (Central Composite Design). The fly ash and sugarcane fiber were ground in a plate mill pulverizer, and the ground fiber was segregated with 5 different wt% levels of CNT fillers/reinforcements to form the CCD matrix. The wt.% values for fly ash and sugarcane fiber ranged from 0 to 2. Similarly, the wt.% values for the MWCNTs were in the range of 0 to 1. The required proportion of epoxy resin, its hardener, fly ash, sugarcane, and CNTs were established by weighing them in the machine. An HY951 hardener was added to the prepared epoxy matrix/reinforcement solution in a proportion of 1:10 by volume. The solution was carefully stirred for around 10 min. After a few minutes, this solution started losing its viscosity, and when it again started to gain consistency, the solution was poured into a tensile mold. It was previously observed that a fly ash (0 to 2 wt.%) and sugarcane fiber (0 to 2 wt.%) with CNTs (0 to 1 wt.%)-reinforced matrix provides the best tensile results [31]. This combination also resists crack propagation and improves fracture toughness. The results of this study showed that there was a remarkable improvement in the mechanical and thermal stability of the composites following reinforcement. 

**Figure 2:**
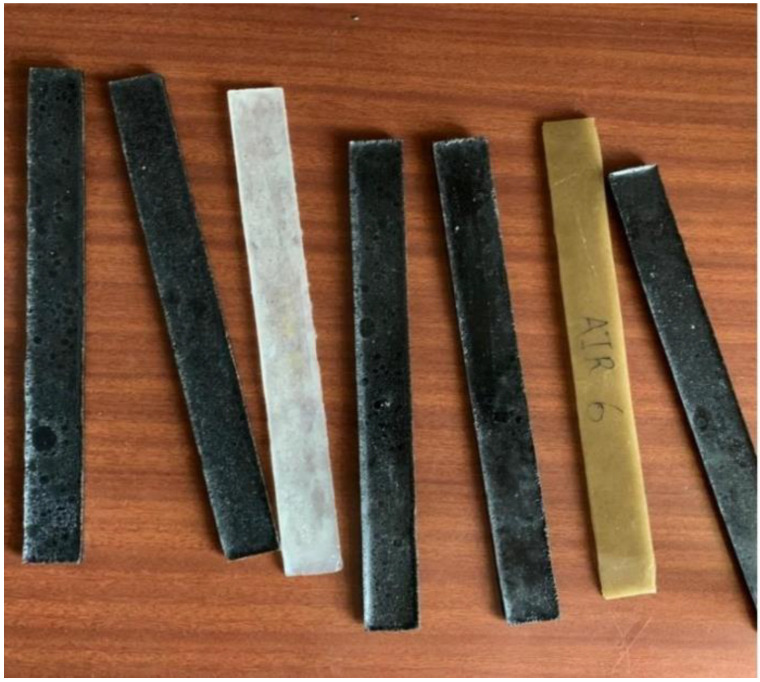
Specimens used for tensile test.

The solution was allowed to solidify in atmospheric conditions for 3–4 h. The involved process was exothermic. Once the polymer matrix composite hardened, it was taken out of the mold. Solidified epoxy matrix composite samples were cured in an oven at 80 ºC for 2 h. A tensile test was carried out with a 50 kN universal testing machine (SHIMADZU make AG-X plus, Shimadzu Asia Pacific, Pvt.Ltd, Chennai, Tamilnadu, India) at a constant displacement rate of 2 mm/min at room temperature (see Figure 2) according to the ASTM: D3039 standard specimen configuration [5]. Generally, a 40 mm length was used from both sides of each sample for them to be held in the holding fixture. The strain rate maintained during the testing was 2 mm/min. Accordingly, 20 samples were prepared for different wt.% of fly ash, sugarcane fiber and CNTs, as listed by the authors in a previous study [31]. 

Equations (1)–(3) are the regression equations for yield stress, UTS, and Young’s modulus, respectively. From these regression equations, values for all 20 samples were calculated by substituting the corresponding wt.% of fly ash/sugarcane fiber/CNTs in Equations (1)–(3). Based on the DOE method, the following regression equations were obtained.

Regression Equation (1):

(1) $$\begin{array}{c} \mathrm{Yield strength}\left(MPa\right)=2.962-3.338\;\mathrm{fly ash}-1.631\;\mathrm{sugarcane}\\ +1.68\;CNT+2.022\;\mathrm{fly ash}\times \mathrm{fly ash}+2.257\;\mathrm{sugarcane}\times \mathrm{sugarcane}\\ -1.971\;\mathrm{CNT}\times \mathrm{CNT}-1.355\;\mathrm{fly ash}\times \mathrm{sugarcane}+0.990\;\mathrm{fly ash}\\ \times \;CNT-0.650\;\mathrm{sugarcane}\times CNT \end{array}$$

Regression Equation (2):

(2) $$\begin{array}{c} UTS\left(MPa\right)=10.99-1.93\;\mathrm{fly ash}-9.76\;\mathrm{sugarcane}+13.72\;CNT+3.969\\ \mathrm{fly ash}\times \mathrm{fly ash}+3.346\;\mathrm{sugarcane}\times \mathrm{sugarcane}+19.86\;CNT\times CNT\;+\\ 2.980\;\mathrm{fly ash}\times \mathrm{sugarcane}-23.29\;\mathrm{fly ash}\times \mathrm{CNT}-2.56\;\mathrm{sugarcane}\times CNT \end{array}$$

Regression Equation (3):

(3) $$\begin{array}{c} \mathrm{Young}’\mathrm{s modulus (MPa)}=537-615\;\mathrm{fly ash}-317\;\mathrm{sugarcane}+401\\ \mathrm{CNT}+341.8\;\mathrm{fly ash}\times \mathrm{fly ash}+392.3\;\mathrm{sugarcane}\times \mathrm{sugarcane}-473\;CNT\;\times \\ CNT-200\;\mathrm{fly ash}\times \mathrm{sugarcane}+196\;\mathrm{fly ash}\times \mathrm{CNT}-128\;\mathrm{sugarcane}\times CNT \end{array}$$

### 2.2. Development of MOEA/D

For optimization, a multi-objective evolutionary algorithm based on decomposition (MOEA/D) was adopted. This is a multi-objective version of genetic algorithm workflow shown in Figure 2. The algorithm’s prerequisites, such as objective functions, number of objectives, population size, ideal vector, and number of neighbors, are first initialized. The initial populations are randomly generated to satisfy the input constraints. An ideal vector is an imaginary solution point in a Euclidean space, and the MOEA/D algorithm tries to generate the ideal vector during each iteration. Objective functions are problem-specific computational procedures where the output of the objective function is a scalar or a vector that needs to be minimized or maximized. A population P may contain n number of individuals. An initial non-dominated solution is found by evaluating the population against the objective function. The Tchebycheff method is used to convert the multi-objective vectors into a scalar quantity. Each of the individuals is evaluated with randomly generated weight vectors to effectively explore the given search space. The evaluated individuals are also compared with their neighbors for a better solution. If a better solution is found, the weight vectors are eliminated and the results are produced.

**Figure 3:**
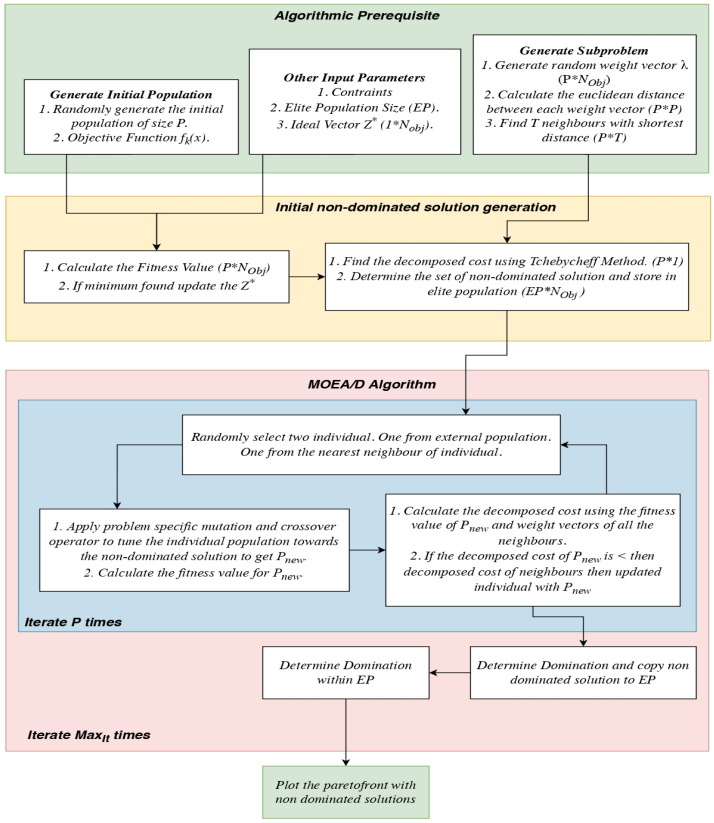
The MOEAD algorithm workflow.

The best-performing solution in the previous population and one more individual are selected using roulette wheel selection. The selected individual undergoes a crossover operation and a mutation operation to generate a new individual. The individual, not satisfying the constraints, is eliminated from the newly developed population. For each newly generated individual, the fitness function is calculated in search of improvements to the solution compared with the previous generation. If any improvement is found, the individual is once again evaluated with the neighbors of different weight vectors for a better solution. The concept of domination is applied to the solution vectors to isolate non-dominated solutions. Non-dominated solutions are the best-performing solutions in all objectives. The non-dominated solutions are stored in the external population (EP) during each outer loop iteration (denoted in blue in Figure 3). The non-dominated solutions are appended in the external population during each iteration. The concept of domination is once again used within the external population to isolate the best-performing individual in the overall procedure. The final solutions of the external populations are plotted in the Euclidean plane. A posteriori expertise is applied to fix the desired solution point among all other solutions. Most MOEAs only regard the MOP as a whole and rely on domination to measure the quality of solutions, and these solutions may not be uniformly distributed over the PF. The fitness evaluation based on a scalar function can be scaled to the number of objectives and strong search ability, and its computational complexity is not exponentially increased with increases in objectives. Therefore, many decomposition-based MOEAs have been proposed.

## 3. Results and Discussions

### 3.1. Tensile Test

The third specimen showed high YS and YM values, whereas the eleventh specimen showed the highest UTS value. Specimen 5 showed the lowest YS and YM values, and sample 12 showed the lowest UTS value. Adding sugarcane and CNTs improved the composite strength, which verified that the CNTs were strong enough to be used as a reinforcement in epoxy composites. The yield strength was low in the 1% fly ash and 1% sugarcane at 1% combination, and it was much higher in the 0% fly ash and 2% sugarcane combination. The increased tensile strength of the composites following the addition of CNTs was due to the fact that CNTs can fill zones in the matrix that cannot be filled by fibers, thus leading to greater interactions between the reinforcement and matrix [39]. In addition, the increase in the tensile strength of the composite was due to the good interaction between the reinforcement, both natural fibers, or CNTs and the epoxy matrix.

### 3.2. Results of Modelling

The predicted models presented a nonlinear relationship between the design parameters and quality characteristics. As reported in a literature review [22], fiber parameters are have significant impact on the quality of products in fiber-reinforced composites. Based on the established relationship models according to the response surface method, the MOEA/D algorithm was applied by considering the multi-objective function as yield strength (YS), ultimate tensile strength (UTS) and Young’s modulus (YM). The initially found objective parameters {YS, UTS, YM} are listed in Table 2 without any constraint on input parameters such as fly ash, sugarcane fiber (SCF) and carbon nanotubes (CNTs). The composite with 2 wt.% of SCF and 0.3854 wt.% of CNTs showed the highest YS and YM values, whereas the composite with 0.0241 wt.% of fly ash, 2 wt.% of SCF, and 1.6714 wt.% of CNTs showed the maximum UTS values. It was noticed that the maximum YS and YM values were achieved with less wt.% of CNTs. On the other hand, the UTS significantly rose with the addition of CNTs, but the composite could not incorporate the fly ash, which is the main problem of disposal. 

Now, to reuse fly ash, {fly ash, SCF, CNT} ≥ 0.2 constraint was applied to the input parameters, and the results obtained regarding {YS, UTS, YM} are illustrated in Table 3. It was found that the composite with 0.2093 wt.% of fly ash, 2 wt.% of SCF, and 0.5780 wt.% of CNTs showed the highest YS and YM values, whereas the composite with 0.2000 wt.% of fly ash, 2 wt.% of SCF, and 1.2829 wt.% of CNTs showed the maximum UTS. The proposed combinations enabled the reuse of fly ash and biodegradable sugarcane fiber, making them environmentally friendly composite materials. 

Considering that more use of fly ash leads to disposal issues, one more case was considered. {Fly ash, SCF, CNT} ≥ 0.5 constraints were applied to input parameters, and the results obtained for {YS, UTS, YM} are shown in Table 4. A combination of 0.5 wt.% of fly ash, 2 wt.% of SCF, and 0.6786 wt.% of CNTs showed the highest YS and YM values, whereas a combination of 0.5273 wt.% of fly ash, 2 wt.% of SCF, and 1.5353 wt.% of CNTs showed the maximum UTS. It was observed that the maximum YS and YM values were obtained when the composite contained the minimum wt.% of fly ash and CNTs, and a maximum wt.% of CNTs led to the highest UTS with a constant wt.% of SCF. 

### 3.3. Confirmatory Test

Table 5 shows the results for {YS, YM} without considering any constraints on {fly ash, SCF, CNT}. It was noticed that constant fly ash and sugarcane fiber contents led to maximum yield strength, Young’s modulus, and UTS values. 

Table 6 illustrates that when {YS and YM} was considered with {fly ash, SCF, CNT} ≥ 0.2 constraints applied to the input parameters, the maximum yield strength and Young’s modulus were obtained for a combination of 0.2 wt.% of fly ash and 2 wt.% of sugarcane fiber, whereas an increase in UTS was noticed following the addition of CNTs up to 0.6095 wt.%. The authors recommend using a small amount of fly ash because it creates many problems during disposal. So, the minimum use of fly ash and the moderate use of SCF and CNTs led to maximum YS and YM values and a sufficient UTS value. One can see that the relative error of each response value was less than 8% in the optimization schemes, so the optimization scheme obtained by combining the response surface method and the MOEA/D algorithm is highly reliable [39]. 

Finally, when {fly ash, SCF, CNT} ≥ 0.5 constraints were considered, the maximum values for objectives {YS, YM} were found as listed in Table 7. A combination of 2 wt.% of sugarcane fiber and 0.5 wt.% of fly ash and CNTs exhibited the maximum yield strength and Young’s modulus values, with a considerable UTS. 

Table 8 shows the wt. content of fly ash/SCF/CNTs obtained with the algorithm and optimization via the RSM. The comparative analysis revealed that the optimal solution of fly ash/SCF/CNTs contents showed a YS of 5.53 MPa and a YM of 914.96 MPa. Based on observations, the projected parametric combination led to the highest yield strength and Young’s modulus values without compromising the percentage of fiber content. Therefore, the design parameters of the scheme were chosen as the optimal parameters. Deviations in the results based on the RSM and MOEA/D were found to be 26.43% and 28.58%, respectively. The results were compared with the experimental results of the optimization by combining the response surface method and the MOEA/D. The combination of 0.2 wt.% of fly ash, 2 wt.% of SCF, and 0.39 wt.% of CNTs showed a maximum YS of 7.52 MPa and a maximum YM of 1281.18 MPa, with a quite considerable UTS of 10.54 MPa, compared with the optimized results obtained through the RSM.

## 4. Conclusions

From the above investigation, the following conclusions are drawn.

- It is evident that fly ash has many environmental disposal issues. Additionally, biodegradable composites can be constructed with the use of SCF and recyclable material such as CNTs.
- A mixture of 0.5 wt.% of fly ash, 2 wt.% of SCF, and 0.5 wt.% of CNTs exhibited better maximum yield strength, Young’s modulus, and UTS values compared with the optimized values obtained via the RSM.
- Increasing the wt.% of CNTs improved the ultimate tensile strength, but it did not influence the composite material’s yield strength and Young’s modulus.
- As the content of fly ash reduced, the YS and YM were increased and the UTS was somewhat degraded; however, making reusable, biodegradable, and recyclable composites necessitates using waste fly ash.
- The MOEA/D is an efficient and effective algorithm that can be used to solve multi-response optimization problems including the one considered in the present study, as it suggests a set of alternate optimal solutions in a short period.
- In this way, the described design parameter combinations are expected to not only ensure the meeting of product quality specifications but also to enhance stability outcomes.

## Figures and Tables

**Table 1:** wt.% of fly ash, sugarcane and CNTs at various levels [31].

Parameters	Levels				
	−2	−1	0	1	2
Wt.% of fly ash	0	0.5	1	1.5	2
Wt.% of sugarcane	0	0.5	1	1.5	2
Wt.% of CNTs	0	0.25	0.5	0.75	1

**Table 2:** Optimizing the objectives {YS, UTS, YM} without any constraints imposed on input parameters {fly ash, SCF, CNT}.

Fly Ash	Sugarcane Fiber	CNT	Yield Strength	UTS	Young’s Modulus
0.0000	2.0000	0.3854	8.5817	11.1184	1457.8263
0.0000	2.0000	0.4394	8.5145	12.4660	1444.6030
0.0000	2.0000	0.4586	8.4877	12.9753	1439.2135
0.0000	2.0000	0.4586	8.4877	12.9753	1439.2135
0.0000	2.0000	0.4626	8.4820	13.0832	1438.0467
0.0000	2.0000	0.4647	8.4789	13.1393	1437.4367
0.0000	2.0000	0.4768	8.4611	13.4691	1433.8081
0.0002	2.0000	0.4889	8.4417	13.8056	1429.8687
0.0000	2.0000	0.5164	8.3986	14.5919	1420.9335
0.0000	2.0000	0.5672	8.3095	16.1206	1402.2802
0.0001	2.0000	0.5728	8.2983	16.2963	1399.9466
0.0002	2.0000	0.5738	8.2959	16.3245	1399.4750
0.0000	2.0000	0.6491	8.1441	18.8052	1367.0065
0.0000	2.0000	0.6501	8.1419	18.8396	1366.5375
0.0002	2.0000	0.6555	8.1290	19.0212	1363.8128
0.0002	2.0000	0.6734	8.0893	19.6476	1355.2231
0.0000	2.0000	0.6815	8.0715	19.9389	1351.3340
0.0000	2.0000	0.7704	7.8509	23.2671	1303.1678
0.0000	2.0000	0.7733	7.8429	23.3808	1301.4316
0.0000	2.0000	0.7865	7.8075	23.9046	1293.6286
0.0000	2.0000	0.8227	7.7065	25.3722	1271.3319
0.0000	2.0000	0.8366	7.6664	25.9492	1262.4478
0.0000	2.0000	0.8713	7.5629	27.4220	1239.4876
0.0000	2.0000	1.0301	7.0278	34.7887	1119.6232
0.0000	2.0000	1.1885	6.3955	43.1286	976.3933
0.0241	2.0000	1.6714	3.7524	73.8677	376.8456

**Table 3:** Optimizing the objectives {YS, UTS, YM} with constraints imposed on input parameters {fly ash, SCF, CNT} ≥ 0.2.

Fly Ash	Sugarcane Fiber	CNT	Yield Strength	UTS	Young’s Modulus
0.2093	2.0000	0.5780	7.2319	14.6589	1224.2724
0.2000	2.0000	0.6566	7.1290	16.9695	1199.8914
0.2010	2.0000	0.7181	6.9935	18.8788	1170.5120
0.2000	2.0000	0.7197	6.9944	18.9424	1170.4461
0.2016	2.0000	0.7205	6.9853	18.9512	1168.8706
0.2000	2.0000	0.7317	6.9670	19.3357	1164.4170
0.2021	2.0000	0.7390	6.9405	19.5532	1159.1280
0.2026	2.0000	0.7397	6.9366	19.5702	1158.4023
0.2019	2.0000	0.7440	6.9297	19.7214	1156.6933
0.2005	2.0000	0.7549	6.9101	20.1045	1152.0019
0.2000	2.0000	0.8001	6.7999	21.6878	1127.4320
0.2000	2.0000	0.8243	6.7365	22.5624	1113.3198
0.2016	2.0000	0.8404	6.6860	23.1372	1102.4820
0.2000	2.0000	0.9602	6.3370	27.9146	1023.6403
0.2000	2.0000	0.9771	6.2821	28.6331	1011.2377
0.2000	2.0000	1.0785	5.9301	33.1704	931.3588
0.2000	2.0000	1.0893	5.8902	33.6772	922.2885
0.2000	2.0000	1.2829	5.0970	43.5611	740.7246

**Table 4:** Optimizing the objectives {YS, UTS, YM} with constraints imposed on input parameters {fly ash, SCF, CNT} ≥ 0.5.

Fly Ash	Sugarcane Fiber	CNT	Yield Strength	UTS	Young’s Modulus
0.5273	2.0000	1.5353	2.8402	49.2434	298.4288
0.5003	2.0000	1.0391	4.9895	26.1341	791.7830
0.5002	2.0000	0.9459	5.2731	22.7455	856.7120
0.5000	2.0000	0.9306	5.3169	22.2262	866.6686
0.5000	2.0000	0.9201	5.3459	21.8733	873.2882
0.5000	2.0000	0.8442	5.5434	19.4455	918.1744
0.5000	1.9998	0.8327	5.5702	19.0966	924.3141
0.5000	1.9963	0.7745	5.6816	17.4055	950.6806
0.5000	1.9983	0.7461	5.7549	16.6386	966.4199
0.5000	2.0000	0.7364	5.7849	16.3898	972.5752
0.5000	2.0000	0.7362	5.7855	16.3829	972.6952
0.5000	2.0000	0.7362	5.7855	16.3829	972.6952
0.5000	2.0000	0.7327	5.7926	16.2907	974.2889
0.5000	2.0000	0.6919	5.8714	15.2614	991.8523
0.5000	2.0000	0.6786	5.8957	14.9396	997.2479

**Table 5:** Optimizing the objectives {YS, YM} without constraints imposed on input parameters.

Fly Ash	Sugarcane Fiber	CNT	Yield Strength	Young’s Modulus	UTS *
0.0000	2.0000	0.1598	8.7384	1483.2924	6.7355
0.0000	2.0000	0.3464	8.6231	1465.6664	10.2167
0.0000	2.0000	0.1535	8.7399	1483.3125	6.6427

* UTS was manually calculated based on resultant optimal input values.

**Table 6:** Optimizing the objectives {YS, YM} with constraints imposed on input parameters {fly ash, SCF, CNT} ≥ 0.2.

Fly Ash	Sugarcane Fiber	CNT	Yield Strength	Young’s Modulus	UTS *
0.3998	2.0000	0.6095	6.3738	1081.4500	14.0438
0.2000	2.0000	0.3984	7.5167	1281.1804	10.5416
0.3296	2.0000	0.4227	6.2962	1076.4423	10.2521

* UTS was manually calculated based on resultant optimal input values.

**Table 7:** Optimizing the objectives {YS, YM} with constraints imposed on input parameters {fly ash, SCF, CNT} ≥ 0.5.

Fly Ash	Sugarcane Fiber	CNT	Yield Strength	Young’s Modulus	UTS *
0.5000	2.0000	0.5000	6.1543	1053.4000	11.3038
2.0000	1.6414	0.6483	4.1821	733.3386	10.1031
1.9946	1.9946	0.7960	5.3122	930.3963	11.0937
0.6780	1.9180	0.9287	4.3665	712.0578	19.6167
2.0000	1.9803	0.6774	5.3314	941.1743	12.0220
2.0000	2.0000	0.5781	5.4256	961.7629	13.4703
2.0000	2.0000	0.6012	5.4264	961.2842	13.1353
0.7813	1.9717	0.8859	4.5504	753.1745	17.4100

* UTS was manually calculated based on resultant optimal input values.

**Table 8:** Comparison of results obtained with experiments, RSM and multi-objective evolutionary algorithm (with ≥0.5 constraints) for maximum responses.

Optimization Using (MOEA/D) Algorithm		Optimization Using RSM	
Fly Ash % (wt.)	Yield Strength (MPa)	Fly Ash % (wt.)	Yield Strength (MPa)
0.2	7.5167	0.5	5.53
Sugarcane fiber % (wt.)	Ultimate tensile stress (MPa)	Sugarcane fiber % (wt.)	Ultimate tensile stress (MPa)
2	10.5416	0.5	19.62
CNT % (wt.)	Young’s modulus (MPa)	CNT % (wt.)	Young’s modulus (MPa)
0.3984	1281.1804	0.5	914.96

## Data Availability

The data presented in this study are available on request from the corresponding author.

## Nomenclature

UTS	Ultimate Tensile Strength
CNT	Carbon Nanotube
YS	Yield Strength
YM	Young’s Modulus
MOEA/D	Multi-Objective Evolutionary Algorithm with Decomposition
DOE	Design of Experiment
ANOVA	Analysis Of Variance
RSM	Response Surface Methodology
SCF	Sugarcane Fiber
EP	External Population
CCD	Central Composite Design
MWCNT	Multi-Wall Carbon Nanotube
PF	Pareto Front
UTM	Universal Tensile Machine
